# Four‐bundle anatomic deltoid ligament reconstruction: Surgical technique

**DOI:** 10.1002/jeo2.70279

**Published:** 2025-09-04

**Authors:** Bruno Olory, Piero Agostinone, Ashraf T. Hantouly, Francesca Zannoni, Khalid Al‐Khelaifi, Emmanouil Papakostas, Alan Getgood, Pieter D'Hooghe

**Affiliations:** ^1^ Aspetar Orthopaedic and Sports Medicine Hospital Doha Qatar

**Keywords:** ankle, deltoid ligament, instability, medial ankle, reconstruction

## Abstract

The deltoid ligament, the strongest medial ankle ligament, is often overlooked in ankle injuries, despite its substantial role in ankle stability. Medial sprains, accounting for 3%–4% of ankle injuries, are frequently misdiagnosed, leading to chronic pain, instability, and hindfoot valgus deformity. While most cases respond to conservative treatment, chronic injuries that fail conservative measures require surgical intervention. This technical paper introduces a novel four‐bundle deltoid ligament reconstruction technique, restoring two anterior (superficial and deep) and two posterior (superficial and deep) bundles. This approach provides a solution for severe injuries and high‐demand patients, permitting functional stability.

**Level of Evidence:**

Level V.

**Abbreviation**
MRI: magnetic resonance imaging

## INTRODUCTION

The deltoid ligament is the strongest ligament on the medial side of the ankle. While most ankle sprains involve the lateral ligament complex, injuries to the medial side are more common than generally believed. They are often neglected, overlooked and undertreated, leading to chronic insufficiency and additional strain on other tissues, particularly the spring ligament [11].

Deltoid ligament sprains are thought to account for ‘only’ 3%–4% of all ankle ligament injuries [15]. They often result from eversion and external rotation of the ankle, leading to medial ankle instability [7, 9].

Most deltoid ligament sprains are treated successfully with conservative measures; it is reasonable to state that chronic medial ankle instability occurs mainly due to poor healing of extensive tears, improper treatments of acute injuries or iatrogenic factors. These chronic injuries can lead to persistent pain, instability, and progressive valgus deformity of the hind foot [8].

Accurate diagnosis of medial ankle instability is crucial for determining the optimal treatment. Clinical assessment, combined with radiographic and magnetic resonance imaging (MRI), is essential for identifying deltoid ligament injuries. In cases of chronic instability, ligament repair is often ineffective, and ligament reconstruction should be considered [2, 14].

Unfortunately, accurately reproducing the deltoid ligament remains a significant challenge due to its complex anatomy, with its anatomical and functional fascicles still not fully understood.

Most reconstructive techniques proposed so far are based on the rebuild of one or two deltoid bundles, covering only partially the area of the ligament. However, in specific conditions such as complete tear and high‐demanding patients a more extensive approach may be required.

In the present paper, we present a technique that reconstructs the deltoid ligament in a four‐bundle fashion (two anterior bundles—superficial bundle and deep bundle—and two posterior bundles—superficial bundle and deep bundle) in order to reproduce it in a highly anatomic way.

## MATERIALS AND METHODS

### Surgical indication

Ideal candidates for this technique are active patients with a complete chronic deltoid ligament rupture confirmed on MRI, such as professional athletes who require higher stability. It may also be considered for patients with chronic non‐reparable deltoid ligament tear, even if their demands are lower, especially in the case of instability associated with acquired flatfoot.

### Operative technique

#### Initial setup and positioning

The patient is positioned supine. A pneumatic tourniquet is applied at the root of the limb. A lateral support is applied to the thigh to maintain stable flexion of the knee during graft harvesting (Gracilis tendon). After skin preparation and sterile draping, the pneumatic tourniquet is inflated.

#### Gracilis tendon graft harvesting

The harvesting of the Gracilis is performed from the ipsilateral knee. Through a vertical approach of 4 cm over the pes anserinus area, about 1 cm medial to the anterior tibial tubercle, after dissection of the subcutaneous tissues, the distal end of the Gracilis is exposed. Adhesions such as vincula are released, and the tendon is stripped. We do not recommend the use of semi‐tendinous because it is too thick to be inserted into a 5‐ or 6‐mm tunnel; in the case of insufficient length of the autograft, a Gracilis allograft is suggested.

**Figure 1:**
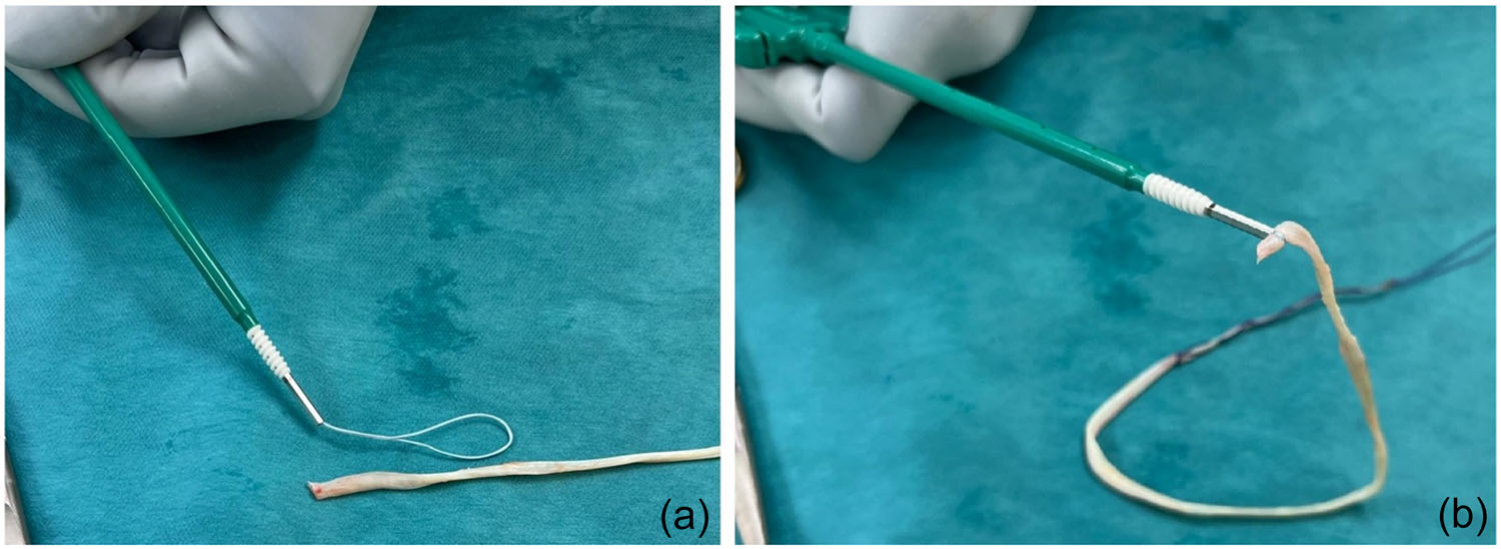
Preparation of the SwiveLock fixed to the graft. A FiberLink is inserted into the cannulated anchor (a). The graft is passed into the suture loop, subsequently pulled to secure the Gracilis to the implant (b).

The thickest end of the graft is secured to a Swive‐Lock 4.75 mm (Arthrex) substituting the inner suture with a FiberLink (Arthrex) (Figure 1).

#### Medial ankle approach

The skin incision is made over the medial side of the distal leg and ankle. It begins approximately 5 cm proximally to the apex of the medial malleolus and extends distally, describing an anterior curve at the level of the ankle to reach the navicular bone. The medial malleolus, the medial aspect of the navicular, the medial aspect of the talus, and the Sustentaculum tali must be exposed through the surgical approach. To obtain optimal visualization of the posterior ankle, direct visualization of the tibialis posterior tendon and its groove should be achieved. Retraction of the tendon posteriorly allows for safe access to the deeper bony structures.

#### Tarsal tunnels preparation

All tunnels are created as blind end half tunnels. The diameter of the tunnels is 5 mm, and the depth is 2 cm. It is recommended to establish the correct direction and position of the tunnels using guide wires that allow for the creation of the definitive half tunnels through over‐drilling with a cannulated driller. To ensure more safety, the use of fluoroscopy might be necessary.

There are two talar tunnels: one anterior and one posterior. The anterior talar tunnel entry point is located on the medial aspect of the talar neck. The direction of this tunnel is posterolateral toward the anterior aspect of the lateral malleolus. The posterior talar tunnel entry point is located anterior to the posteromedial talar tubercle, just underneath the talar superior articular surface. The direction of this tunnel is anterolateral toward the sinus of tarsi. In this way, both tunnels are developed in the body of the talus with convergent directions. To reduce the risk of tunnels coalitions, it is suggested to check the direction of the two guide wires before drilling with the 5 mm perforator. An angle slightly less than 90° is recommended between the two wires.

#### Navicular tunnel preparation

The navicular half tunnel is created in the posterior part of the bone's medial aspect, following the transverse axis of the navicular.

#### Calcaneal tunnel preparation

The calcaneal tunnel entry point is located at the level of the superior part of the Sustentaculum tali and directed postero‐inferiorly following the shape of the bony prominence, to avoid breaking the Sustentaculum or damaging the subtalar joint.

#### Tibial tunnels preparation

To create the two tibial tunnels, two 4 mm pin wires are necessary. A first pin wire is introduced from the intercollicular groove of the medial malleolus toward the tibialis tendon groove. The direction is determined to obtain a distance of approximately 5 cm from the apex of the malleolus (posterior tunnel). A second pin wire is inserted 1 cm proximally to the first one (proximally to the anterior colliculus) with a parallel or slightly convergent direction (anterior tunnel). Both tunnels should be at least 3 cm in length. Once the pin wires are in place, 6 mm diameter sockets are drilled for 20 mm. The pin wires should be provided with a buttonhole to retrieve one transport suture for each tunnel.

**Figure 2:**
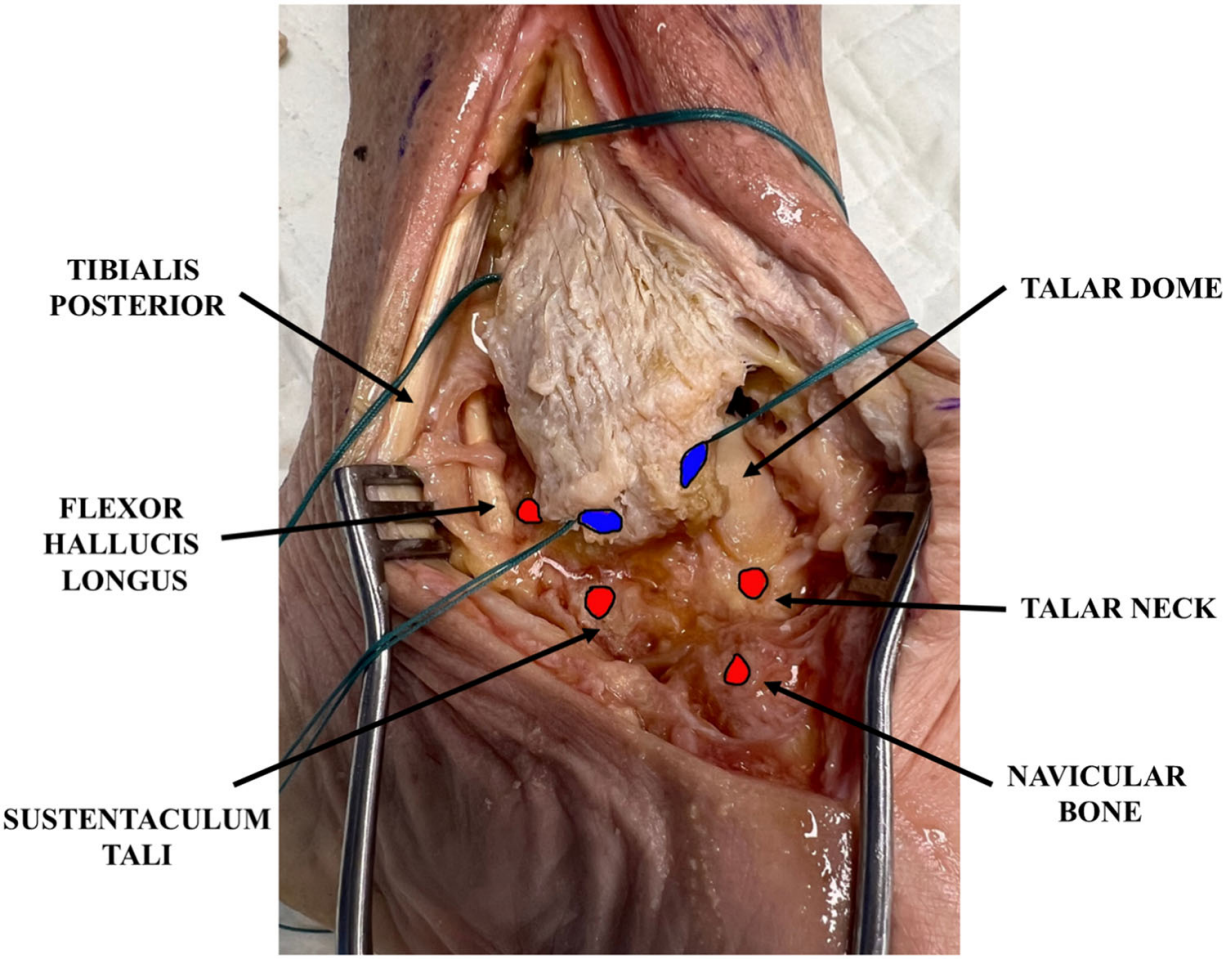
The anatomic landmarks and the entry points of the six tunnels (four talar in red and two tibial in blue).

Figure 2 shows the anatomic landmarks and entry points of the six tunnels (four talar in red and two tibial in blue).

#### Graft passage and tensioning

The graft positioning begins with the anterior talar tunnel. The thickest portion of the graft (usually the distal one) is inserted and fixed in the socket using the previously prepared SwiveLock 4.75 mm (Arthrex). The distance of the anterior tibio‐talar bundle is assessed and marked on the graft (skin marking pen). A further mark is drawn 3 cm beyond. An UltraButton Adjustable (Smith & Nephew) is used to wrap the graft and is positioned between the two marks. The button is subsequently pulled into the anterior tibial tunnel and used to retrieve the graft until the two marks are at the level of the entrance of the tunnel. Then, the graft is directed to the navicular socket, and a mark is drawn to assess the distance between the tibia and the entrance of navicular half tunnel. A second SwiveLock with FiberLink is secured to the graft 2 cm beyond the last mark and used to provide the final fixation. Final tensioning is provided through the adjustable cortical button with the foot in resting position.

The remaining graft is cut and measured. To ensure the reconstruction of the two posterior bundles, the length should be at least 11–12 cm. Otherwise, a Gracilis tendon allograft is necessary.

The reconstruction of the posterior bundles follows the same steps as the anterior ones, starting from the sustentaculum tali tunnel. The fixation in the tarsal tunnels is provided by two Swive‐Locks with FiberLink, and the posterior tibial tunnel fixation is guaranteed by an Ultrabutton Adjustable. In this case, the final tensioning must be performed with the foot in a neutral position.

All the steps of the reconstruction are summarized in Table 1.

**Table 1:** Step‐by‐step description of the deltoid ligament reconstruction.

Steps	Brief description	Images
Skin incision	It should start 5 cm above the medial malleolus and extend distally, curving anteriorly to reach the navicular bone.	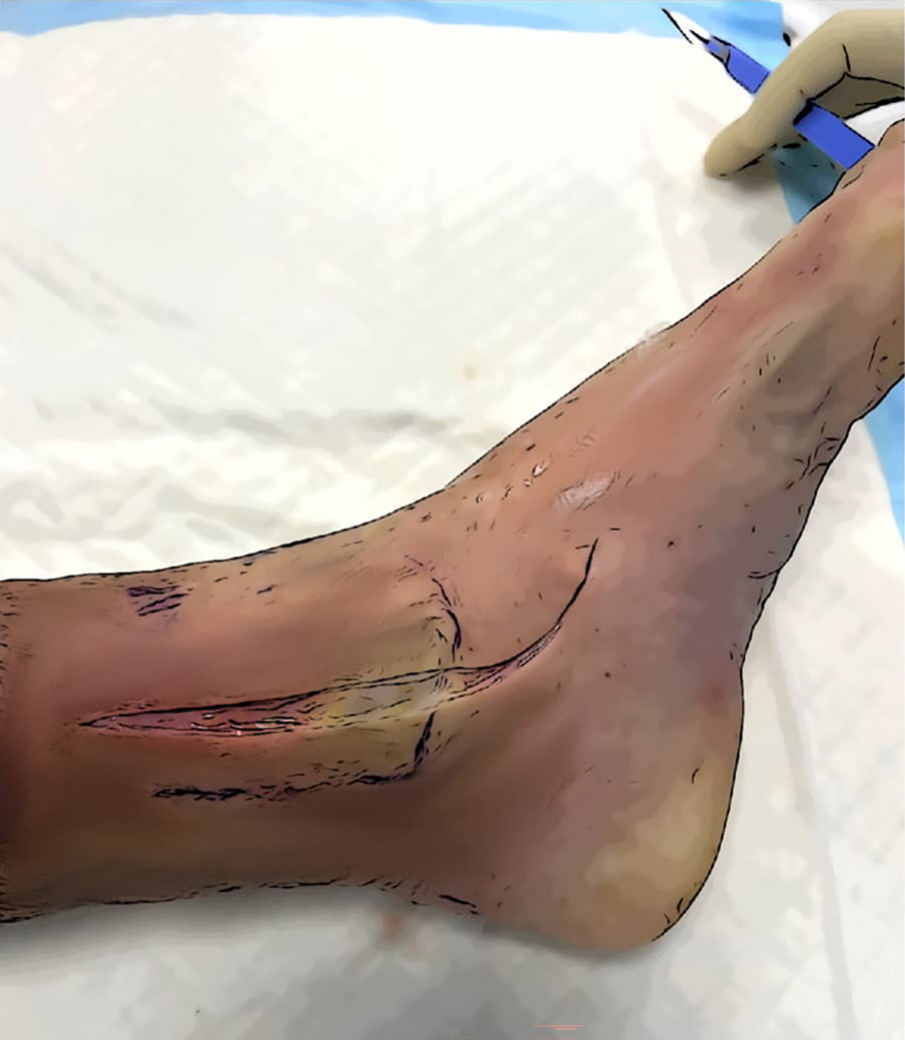
Surgical exposure	The medial malleolus, the medial aspect of the navicular, the medial aspect of the talus, and the Sustentaculum tali must be exposed. Direct visualization of the tibialis posterior tendon and its groove is needed. The posterior retraction of the tendon allow the protection of the posterior neurovascular structures of the tarsal tunnel.	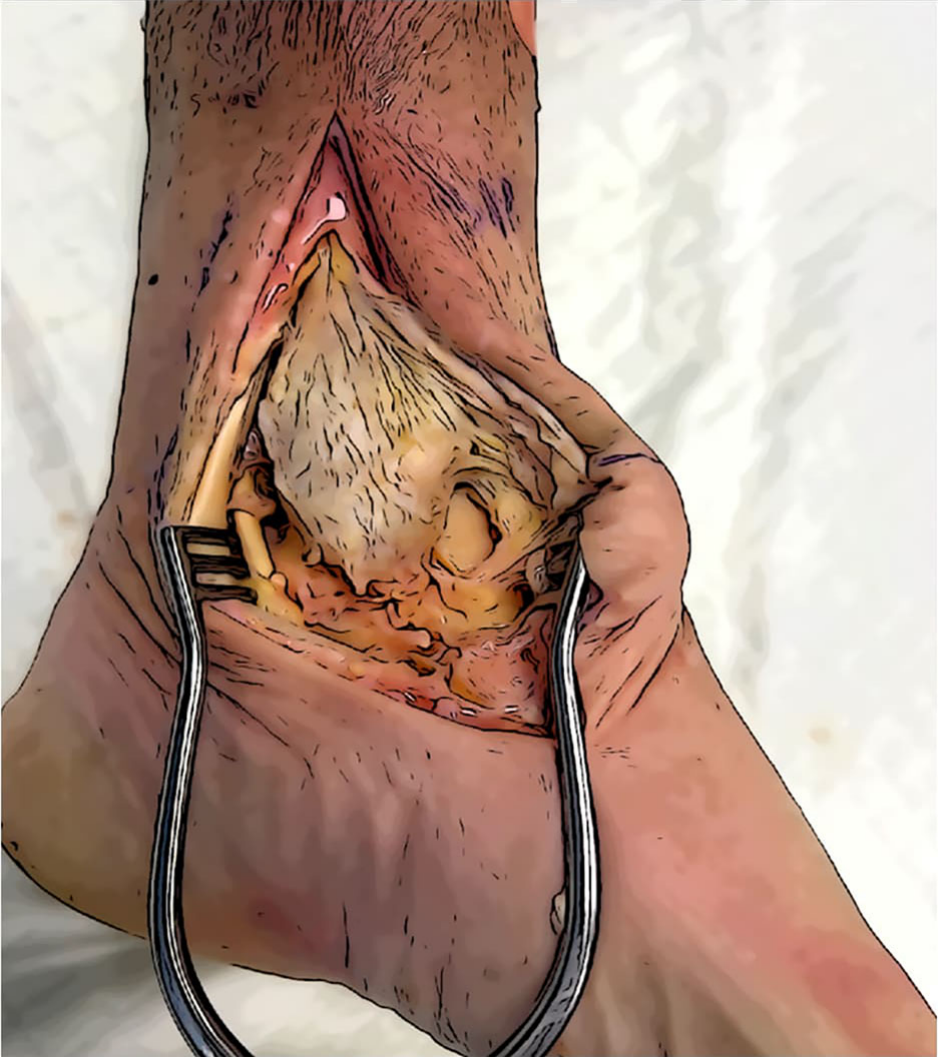
Talar tunnels	Sockets 5 mm in diameter and 2 cm deep. Anterior tunnel from talar neck extends directly posteriorly to the anterior aspect of the fibula. Posterior tunnel next to the postero‐medial talar process, directed anterolateral to the Sinus tarsi. The angle between the guide wires should be slightly less than 90°.	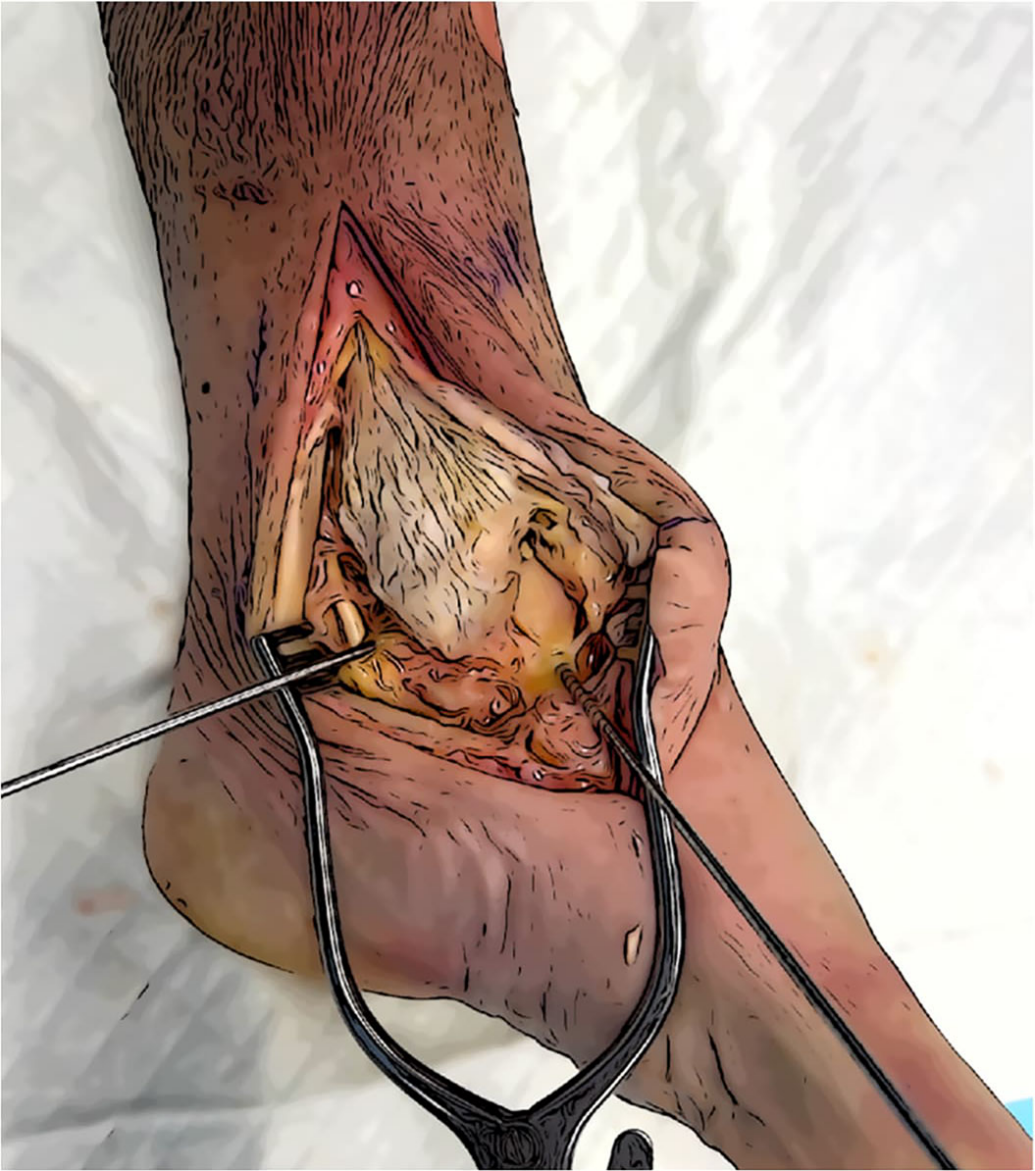
Navicular and Calcaneal Tunnels	Navicular tunnel entry point is located in the posterior part of the medial aspect, drilled following the transverse axis of the bone. Calcaneal tunnel entry point is located in the Sustentaculum tali, drilled in slightly postero‐inferior direction.	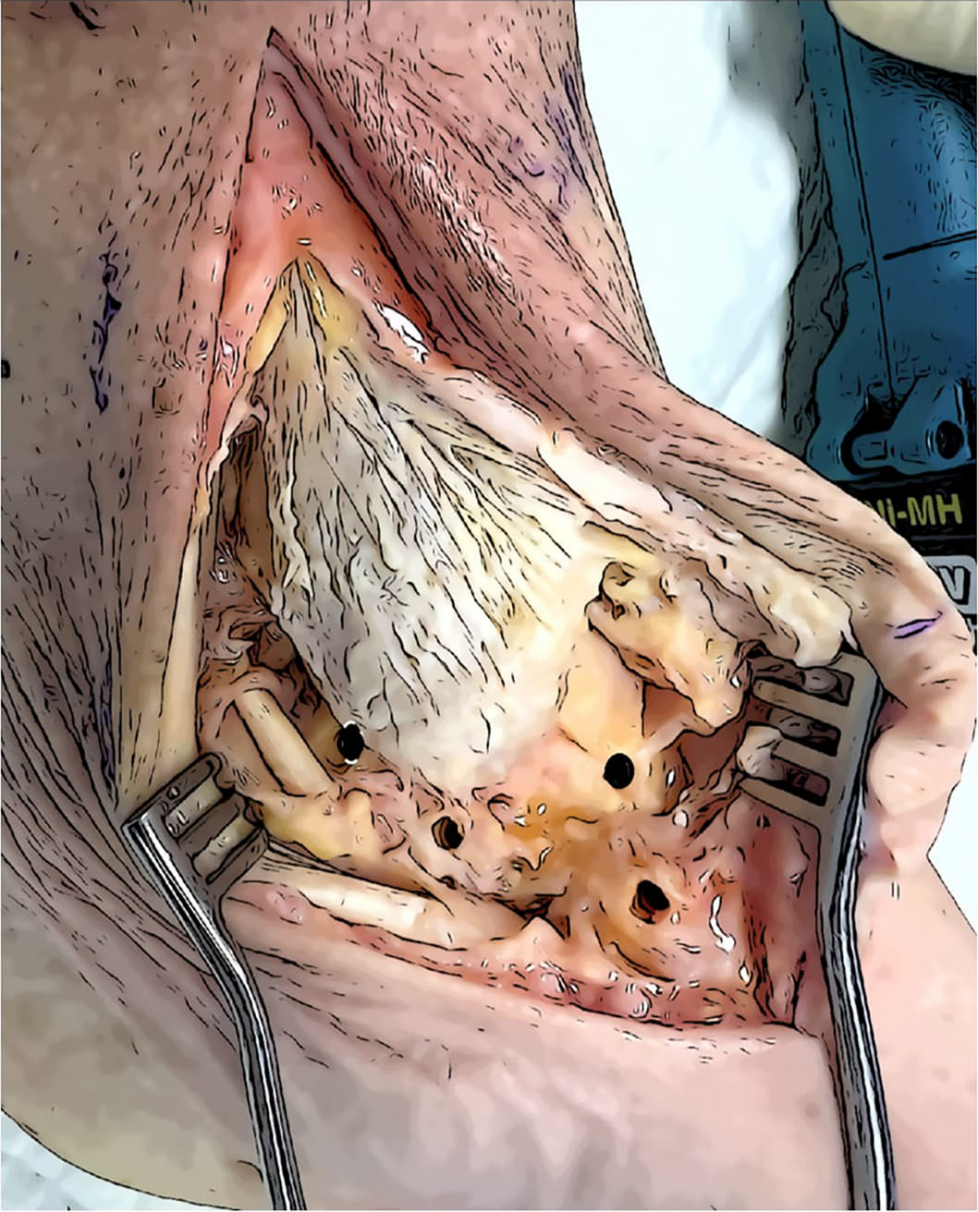
Tibial tunnels	Two pin wires 4 mm with buttonhole, over drill a socket 6 mm in diameter and 2 cm deep for each one. Posterior tunnel at the tip of the medial malleolus going to the tibialis groove 5 cm from the apex. Anterior tunnel 1 cm proximal to the first one. The complete tunnels should be parallel and at least 3 cm long.	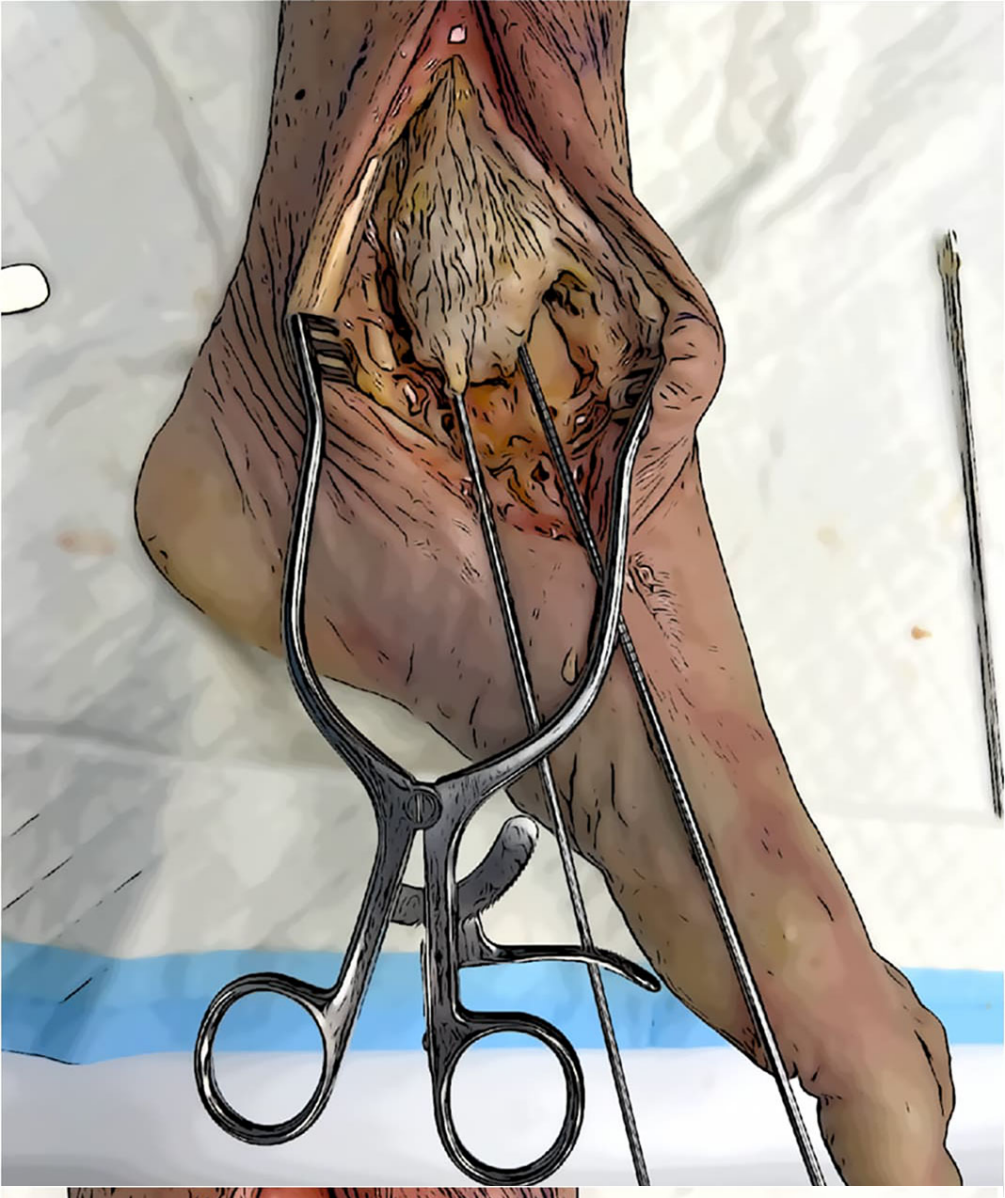 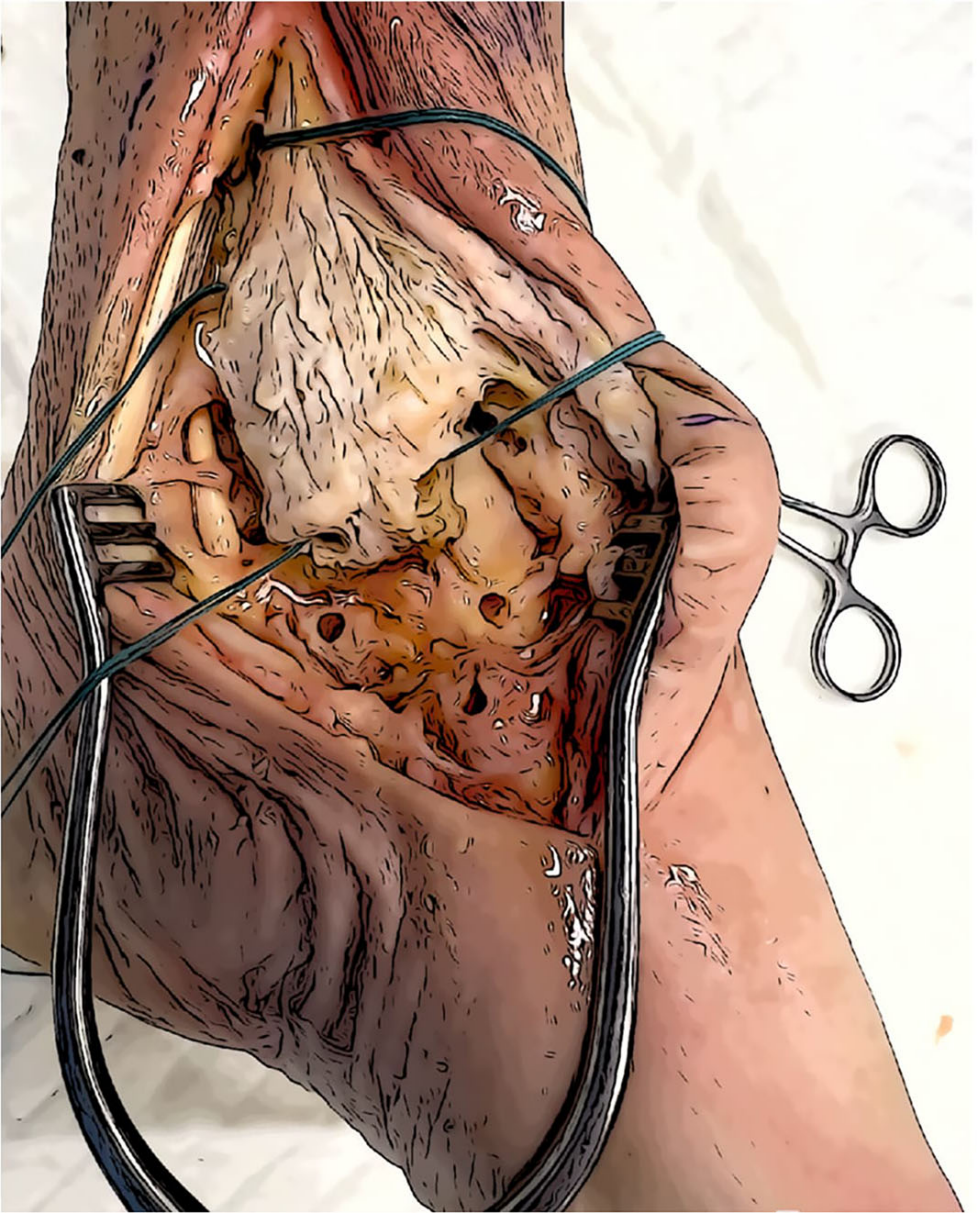
Anterior bundles reconstruction	Anterior talar fixation with a SwiveLock, the approximate length from the anterior talar tunnel to the anterior tibial tunnel is measured and marked, and a second mark is drawn 3 cm beyond.	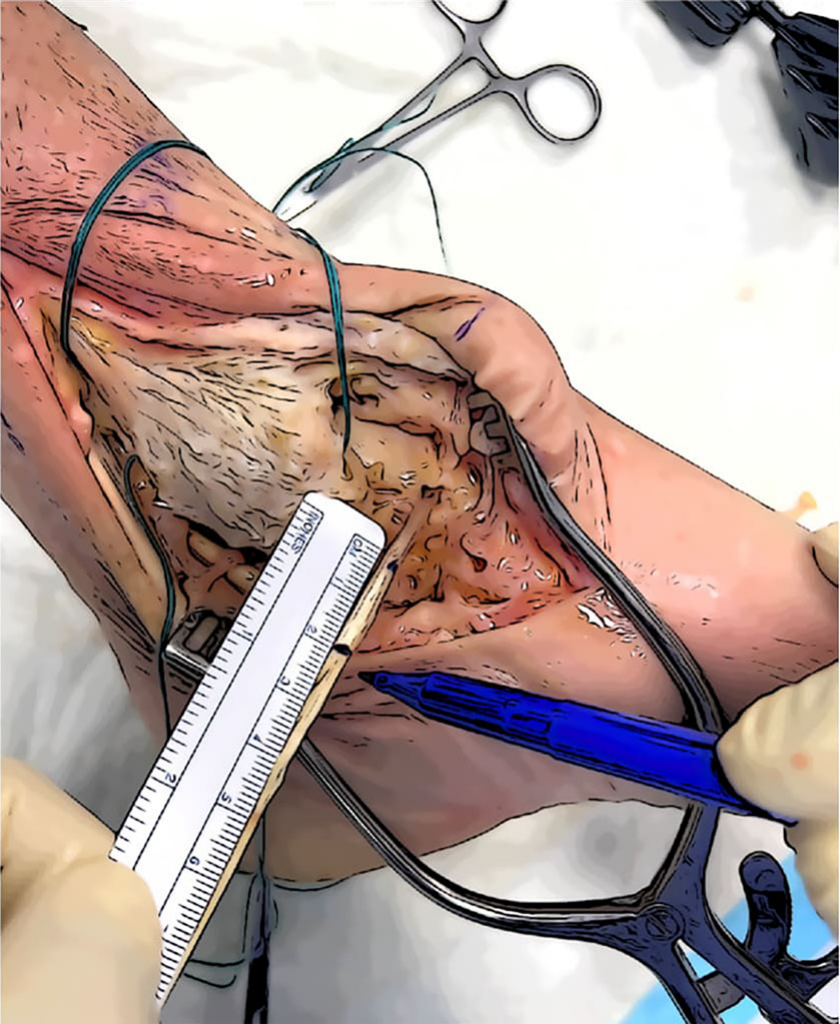
	An UltraButton is positioned between the two marks, pulled through the anterior tibial tunnel, and used to retrieve the graft until the two pen marks are at the entrance.	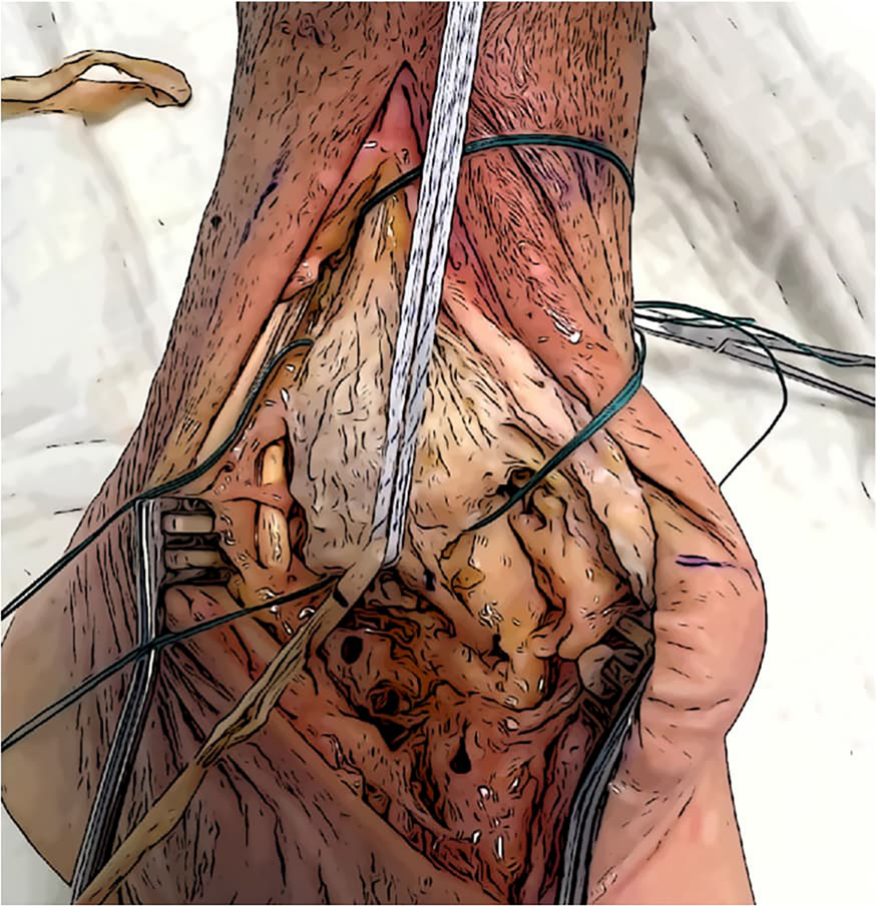
	The length from the anterior tibial tunnel and the navicular one is assessed and marked on the graft. Navicular fixation with a second SwiveLock secured 2 cm beyond the mark. Final tensioning with the foot in rest position. Cutting the remnant and assessment of the length (at least 11–12 cm).	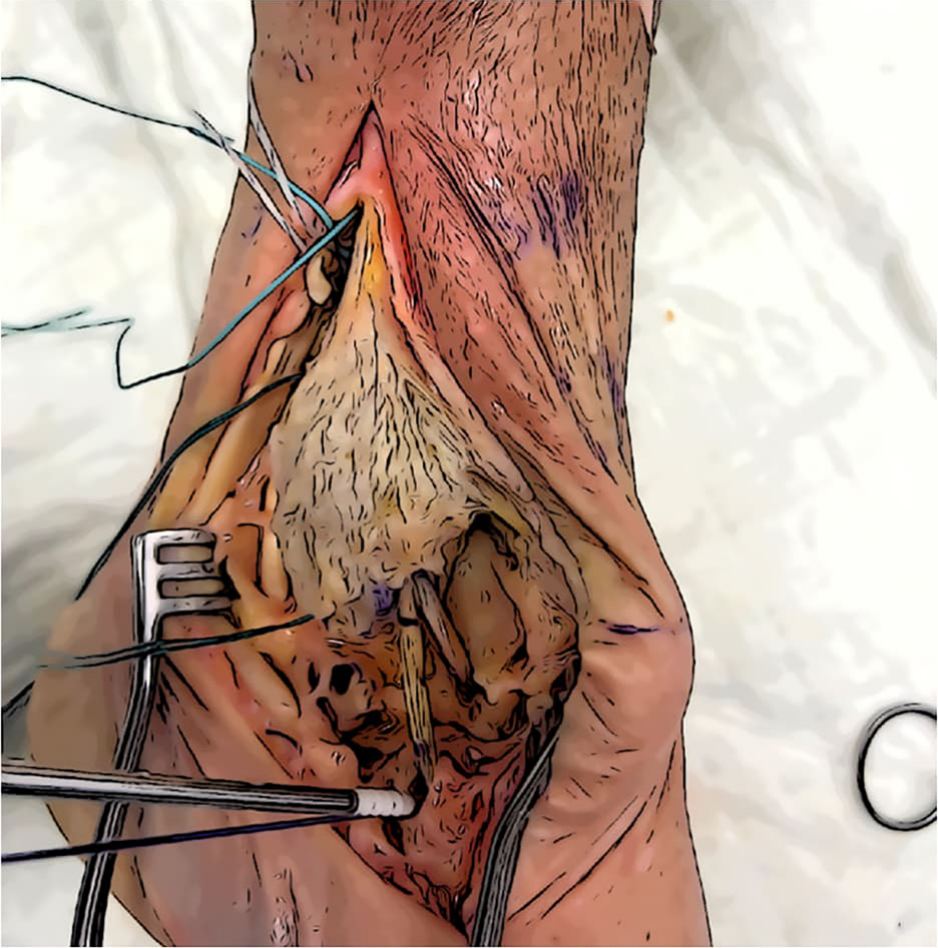
Posterior bundles reconstruction	Remnant fixation to the Sustentaculum with a SwiveLock, the approximate length from the Sustentaculum to the posterior tibial tunnel is measured and marked, and a second mark is drawn 3 cm beyond.	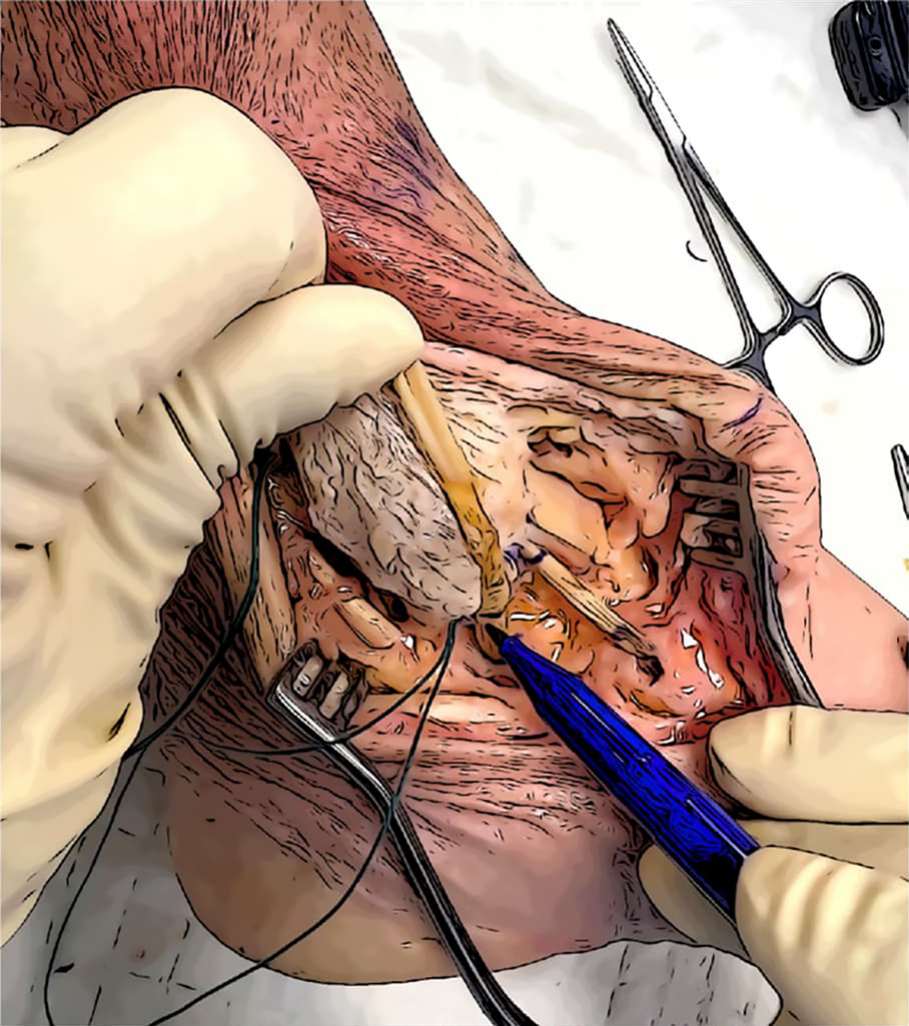
	An UltraButton is positioned between the two marks, pulled through the posterior tibial tunnel, and used to retrieve the graft until the two pen marks are at the entrance.	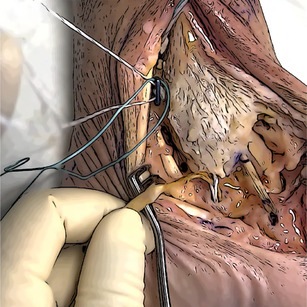
	The length of the posterior tibio‐talar bundle is marked on the graft, fixation into the posterior talar tunnel with a second Swivelock secured 2 cm beyond the mark. Final fixation and tensioning with the foot in neutral position.	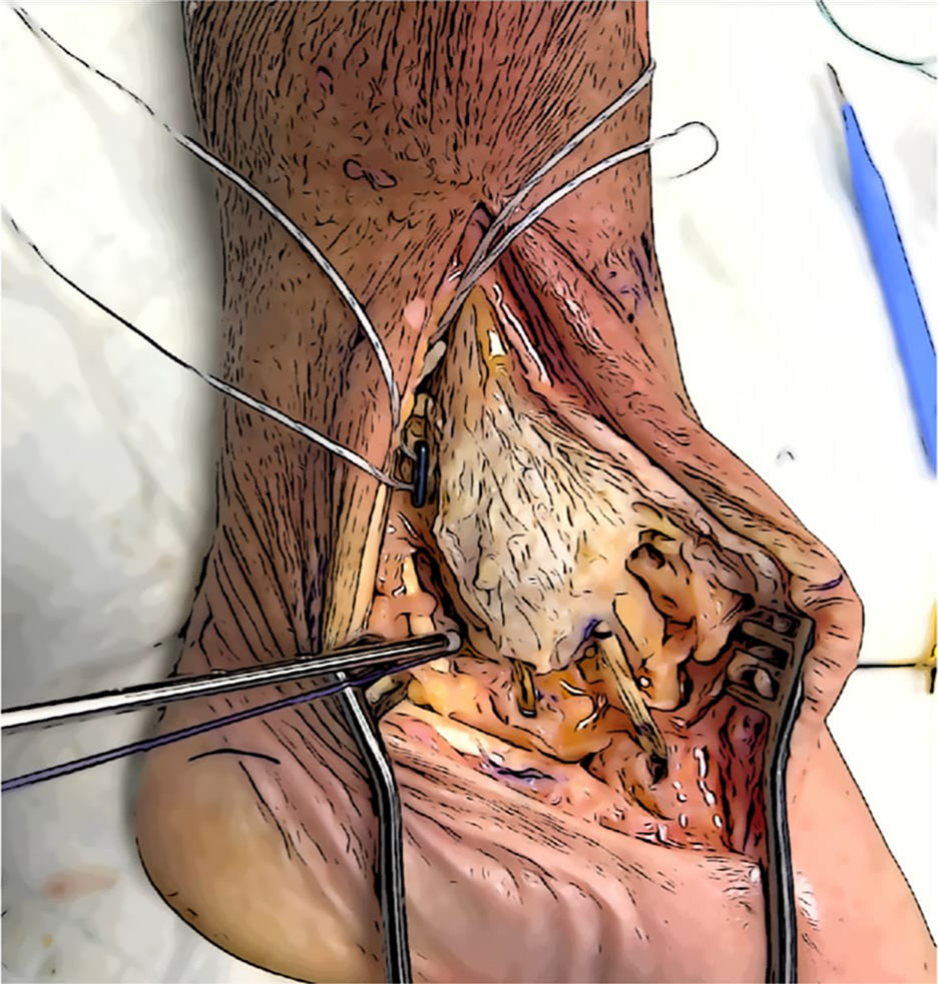
Final result	The deltoid ligament is reconstructed in a four‐bundle fashion. After the closure of the surgical wound, the limb is secured in a walking boot.	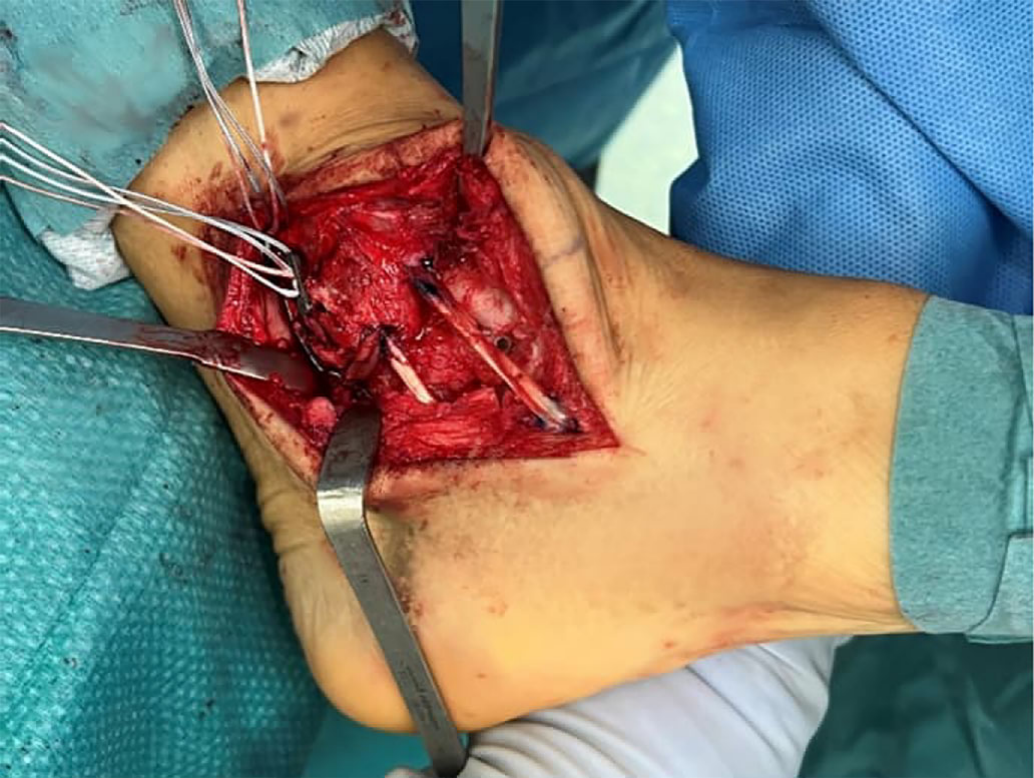

### Postoperative treatment

After surgery, the operated limb is immobilized in a walking boot for 6 weeks. Full weight‐bearing is immediately permitted. In cases of flatfoot, or hindfoot valgus an insole supporting the medial arch is suggested to avoid graft elongation and overload.

Rehabilitation begins on the eighth day. The first 6 weeks focus on providing relief for postoperative inflammation and recovering full range of motion (avoiding foot pronation). Strengthening exercises of the ankle inversion and dorsiflexion (tibialis anterior and extensor hallucis longus) can start at the same time.

After 6 weeks, walking is permitted wearing sports shoes and the insole in cases of flatfoot. At that time, running in a straight line, cord jumping, and cautious balance board exercises are allowed, as well as progressive global strengthening of the limb.

At the third month, the patient should be able to run in changing directions.

## DISCUSSION

In the present paper, we are proposing a novel surgical technique for the treatment of chronic medial ankle instability. This technique has been developed at our institution as an advanced treatment option for athletes experiencing medial ankle instability. It builds on our successful experience with lateral ankle ligament reconstruction using the gracilis tendon, which has demonstrated excellent outcomes and a quick return to sports activities [12].

Nowadays, there is few evidence available to guide the management of deltoid injuries without fracture. Acute isolated superficial deltoid injuries without instability may be treated with a short period of immobilization and rehabilitation. Treatment of complete injuries of both deep and superficial deltoid is still debated [17].

However a pure soft tissue injury can lead to significant time away from sport and an early stress through a completely disrupted ligament may lead to healing in stretched position and possibly result in long‐term medial instability [5].

Neglected deltoid ligament injuries can lead to progressive deformity, posterior tibialis tendon dysfunction, flatfoot deformity and a valgus hindfoot.

Patients who fail to become asymptomatic with conservative measures and present with persistence of clinical instability are usually considered for operative treatment. Repair or reconstruction technique of deltoid ligament injury depends on the extent and level of the ligament injury, and several surgical techniques have been proposed [1, 4, 7, 13, 16]

Reconstruction techniques are primarily designed to address chronic cases associated with acquired flatfoot deformity. These methods typically involve the use of peroneus longus or tibialis posterior grafts, and, in rare instances, the application of tapes [4, 7, 11, 13]. In our view, an advantage of our technique lies in utilizing a graft external to the primary surgical site. This approach helps preserve the native anatomy of the joint being treated to the greatest extent possible.

The huge variety of surgical options is also based on inconsistent evidence about the deltoid complex anatomy. Although many studies have been conducted, the anatomy of the ligament remains unclear, with a number of described fascicles up to sixteen units [3].

This is a problem for clinicians when reporting these injuries and for surgical repair and reconstruction procedures, as the lack of detailed description creates uncertainty about which part of the ligament is being repaired or reconstructed and complicates comparison between studies.

Nonetheless, despite the varying number and frequency of deltoid fascicles, the primary agreement in the literature is that the deltoid ligament is structured in six fascicles arranged on two layers: one superficial and one deep [3, 10].

Given its triangular shape, the deltoid ligament's anterior portion is taut in plantar flexion, whereas its posterior portion is taut in dorsiflexion [6]. A recent study by Dalmau‐Pastor et al. [3] proposed a simplified arrangement of the deltoid ligament bundles, identifying four fascicles: three superficial and one deep, fan‐shaped fascicle. Our technique aligns closely with the anatomy described in their study, with two superficial bundles reproducing the three superficial fascicles and two deep bundles restoring the broad, deep fascicle of the native ligament.

Considering the factors mentioned above, the aim of this surgical procedure is to reconstruct the deltoid ligament in the most anatomically accurate manner. Despite the functional nature of the reconstruction, this surgical approach provides the ankle with enhanced strength and stability, allowing it to resist significant stress and let athletes have a quicker return to sport.

Table 2 provides a comprehensive list of pearls and pitfalls of the technique described in the present article.

**Table 2:** Pearls and pitfalls of the technique described in the present article.

Pearls	Pitfalls
Highly anatomic	Risk of damage to structures posterior to the medial malleolus (blood vessels, nerves and tendons)
No need for torn ligament remnants (useful for complete destruction)	Risk of tendinitis for button malpositioning in the malleolar groove
Graft external to the primary surgical site	Risk of talar sockets coalitions
Separate tensioning of posterior and anterior bundles (but caution in not overtightening)	Risk of sustentaculum tali fracture

## AUTHOR CONTRIBUTIONS

All authors made substantial contributions to the study's design. Bruno Olory developed the surgical technique. Bruno Olory, Piero Agostinone, Ashraf T. Hantouly and Francesca Zannoni participated in manuscript drafting. Bruno Olory, Khalid Al‐Khelaifi, Emmanouil Papakostas, Alan Getgood and Pieter D'Hooghe reviewed the manuscript and supervised the whole project. Bruno Olory, Piero Agostinone, Ashraf T. Hantouly and Francesca Zannoni prepared the final version of the manuscript, which was reviewed by all authors.

## CONFLICT OF INTEREST STATEMENT

The authors declare no conflicts of interest.

## ETHICS STATEMENT

The ethics statement is not available.

## Data Availability

Data Availability Statement is not available.
